# The RANKL-RANK Axis: A Bone to Thymus Round Trip

**DOI:** 10.3389/fimmu.2019.00629

**Published:** 2019-03-29

**Authors:** Cristina Sobacchi, Ciro Menale, Anna Villa

**Affiliations:** ^1^Milan Unit, Institute for Genetic and Biomedical Research (CNR-IRGB), Milan, Italy; ^2^Humanitas Clinical and Research Center IRCCS, Rozzano, Italy; ^3^San Raffaele Telethon Institute for Gene Therapy (SR-Tiget), IRCCS San Raffaele Scientific Institute, Milan, Italy

**Keywords:** osteoclasts, denosumab, thymus, central tolerance, rheumatoid arthritis, osteoporosis, tumor

## Abstract

The identification of Receptor activator of nuclear factor kappa B ligand (RANKL) and its cognate receptor Receptor activator of nuclear factor kappa B (RANK) during a search for novel tumor necrosis factor receptor (TNFR) superfamily members has dramatically changed the scenario of bone biology by providing the functional and biochemical proof that RANKL signaling via RANK is the master factor for osteoclastogenesis. In parallel, two independent studies reported the identification of mouse RANKL on activated T cells and of a ligand for osteoprotegerin on a murine bone marrow-derived stromal cell line. After these seminal findings, accumulating data indicated RANKL and RANK not only as essential players for the development and activation of osteoclasts, but also for the correct differentiation of medullary thymic epithelial cells (mTECs) that act as mediators of the central tolerance process by which self-reactive T cells are eliminated while regulatory T cells are generated. In light of the RANKL-RANK multi-task function, an antibody targeting this pathway, denosumab, is now commonly used in the therapy of bone loss diseases including chronic inflammatory bone disorders and osteolytic bone metastases; furthermore, preclinical data support the therapeutic application of denosumab in the framework of a broader spectrum of tumors. Here, we discuss advances in cellular and molecular mechanisms elicited by RANKL-RANK pathway in the bone and thymus, and the extent to which its inhibition or augmentation can be translated in the clinical arena.

## Introduction

Receptor activator of nuclear factor kappa B (RANK) and its ligand (RANKL), encoded, respectively, by the *Tumor necrosis factor receptor superfamily member 11A* (*Tnfrsf11a*) and the *Tumor necrosis factor ligand superfamily member 11* (*Tnfsf11*) genes, constitute a receptor-ligand pair initiating a signaling pathway of paramount relevance in many pathophysiological contexts (1). They have been described in the context of T cell-dendritic cell interactions (2), in bone and in the immune system (3, 4), thus triggering the start of the osteoimmunology era. This axis has revealed an unexpected role in the thermoregulation by the central nervous system (5) and in mammary epithelium development during pregnancy and progesterone-driven breast cancer (3, 6). The RANKL-RANK axis has also been involved in diverse immune-mediated diseases affecting the bone (7–9) as well as other tissues (10), and in cancer settings (11). Overall, this pathway has emerged as a potential target of therapy in a wide range of conditions; which at the same time implies monitoring many different physiological functions when interfering with this axis.

**Figure 1:**
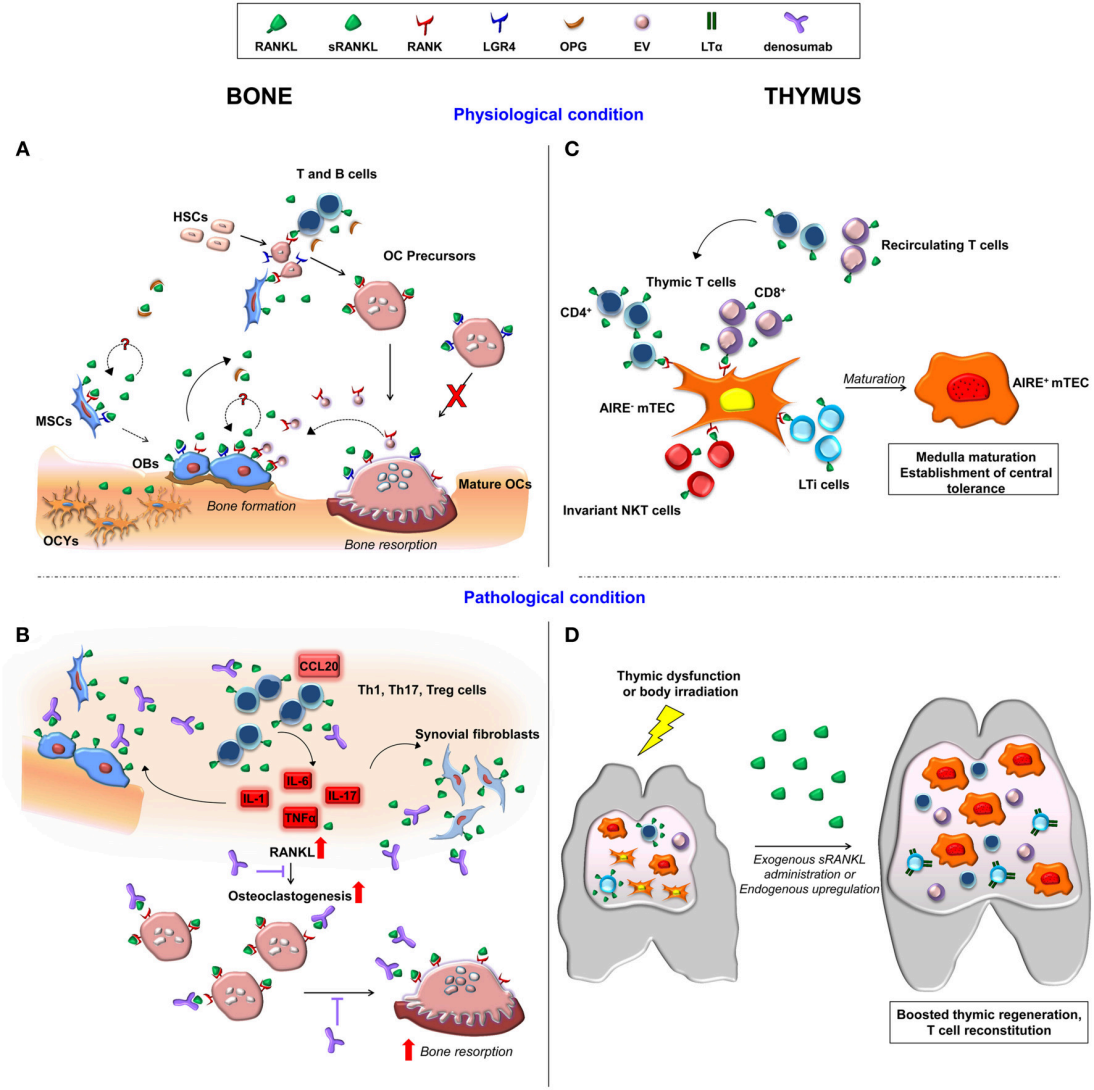
Schematic representation of cellular and molecular players involved in RANKL-RANK signaling axis in the bone and thymus in physiological and in pathological conditions. **(A)** Membrane-bound and soluble RANKL produced by cells of the osteoblast lineage and by immune cells induce osteoclastogenesis upon its binding to RANK on osteoclast precursors. OPG is the soluble decoy receptor for RANKL. Moreover, RANKL binding to LGR4 on osteoclasts hinders their maturation. RANK expression by MSC and osteoblasts points to a potential RANKL autoregulatory mechanism affecting bone formation. In addition, osteoclast-derived RANK-expressing extracellular vesicles (EV) trigger a reverse signaling on osteoblast. **(B)** The inflammatory bone environment in pathological condition, such as osteoporosis and rheumatoid arthritis, results in increased production of RANKL by immune cells, osteoblastic cells and synovial fibroblasts. This exacerbates osteoclast generation and bone loss, which are target of denosumab treatment. **(C)** In the thymus, RANKL produced by resident and recirculating T cells, invariant NKT and LTi cells fosters mTEC AIRE expression and maturation via RANK receptor, allowing correct establishment of central tolerance. **(D)** In the presence of thymic dysfunction, pharmacological sRANKL administration boosts thymic regeneration, and T cell reconstitution. Similarly, in the early phases of thymic regeneration after body irradiation, CD4^+^ and LTi cells upregulate RANKL. This results in increased expression of LTα in LTi cells. OBs, osteoblasts; OCs, osteoclasts; OCYs, osteocytes.

As schematically depicted in Figure 1, here we focus on advances in cellular and molecular mechanisms elicited by RANKL-RANK signaling in two functionally related compartments: the bone and the thymus. Moreover, we review novel perspectives to translate inhibition or enhancement of this pathway in the clinic.

## RANKL-RANK Axis in the Bone

The identification of RANKL-RANK signaling in bone represents a milestone in bone biology (12, 13). Its indispensable role in osteoclast formation is clearly demonstrated by the complete absence of osteoclasts in the *Rankl*^−/−^ and *Rank*^−/−^ murine models (3, 4, 14, 15), as well as in their human counterpart, i.e., patients affected by RANKL-deficient and RANK-deficient osteoclast-poor Autosomal Recessive Osteopetrosis (16, 17). Nonetheless, the possibility of RANKL-independent osteoclastogenesis, particularly in pathologic conditions, has been a matter of a long-lasting debate (18–22) and a general consensus in the field has not been reached, yet.

RANKL is mainly produced by stromal cells in bone, in normal conditions, and primarily by osteocytes (23–25). RANKL is mostly membrane-bound and can be shed to form a soluble protein; the former is sufficient for most functions, while the latter contributes to physiological bone remodeling, as recently demonstrated in mice expressing a sheddase-resistant form of RANKL (26).

The membrane functional receptor RANK is mainly expressed by cells of hematopoietic origin, including also osteoclasts and their precursors, and has been recently detected also in Mesenchymal Stem Cells (MSCs) (27, 28), raising the intriguing hypothesis of an autocrine/paracrine loop in these cells (Figure 1A).

The RANKL-RANK signaling pathway in the osteoclast lineage comprises a plethora of molecules (29). Essentially, upon engagement by its ligand, RANK recruits a number of adaptors (most importantly, TNF Receptor-Associated Factor 6, TRAF6) (30), which converge on kinases activation, including Phosphoinositide-3-Kinase (PI3K) and Mitogen Activated Protein (MAP) kinases. This promotes nuclear translocation and activation of transcription factors, Nuclear Factor of Activated T cell 1 (NFATc1) (31), c-fos (32), and Nuclear Factor kappa B (NF-κB) (33), comprising the master regulator of the osteoclast-specific transcriptional program. The RANKL-RANK pathway interacts with costimulatory signals from immunoreceptor tyrosine based activation (ITAM)-motif containing proteins, further regulating NFATc1 activation (34, 35).

RANKL signaling during osteoclastogenesis results in the generation of reactive oxygen species (ROS), which further stimulate osteoclast formation and bone resorption (36). On the other hand, a variety of antioxidant mechanisms monitors ROS levels and the reciprocal control between these opposite functions (i.e., ROS production and scavenging) importantly impacts on bone homeostasis (37–39).

The RANKL-RANK axis is counterbalanced by the soluble decoy receptor osteoprotegerin (OPG) (40), which is itself controlled by many ligands, including the TNF-Related Apoptosis Inducing Ligand (TRAIL), von Willebrand factor (vWF), and glycosaminoglycans (GAGs) (41). Moreover, the Leucine-rich repeat-containing G protein-coupled receptor 4 (LGR4) is an additional membrane receptor for RANKL, competing with RANK for ligand binding and negatively regulating osteoclastogenesis through the inhibition of NFATc1 activation (42). LGR4 acts also as an R-spondin receptor in bone marrow MSCs and has been recently demonstrated as a key molecule in mesoderm-derived tissue development and MSC differentiation (43), whether RANKL might be involved in this specific context has to be investigated (Figure 1A).

The recognition of the crucial role of RANKL-RANK signaling in osteoclast biology led to the development of the anti-RANKL antibody denosumab, a fully human Immunoglobulin (Ig) G2 monoclonal antibody with high affinity and specificity for human soluble and membrane-bound RANKL (44). Specifically, denosumab binds to the DE loop region of the ligand, which is one of the surface loop structures interacting with the functional receptor on responding cells (44). Denosumab is used as an antiresorptive drug for diverse indications, such as osteoporosis (45), primary bone tumors (46), and osteolytic bone metastases (47). Its use is under evaluation also in other fields, such as solid tumors (11) and Rheumatoid Arthritis (48), and has been very recently proposed in the prevention of BRCA1-associated breast cancer (49). Finally, denosumab administration has been considered in the field of rare diseases too, for example for the treatment of persistent severe hypercalcemia after hematopoietic stem cell transplantation in patients affected by Autosomal Recessive Osteopetrosis (50), in patients affected by Fibrous Dysplasia (51), or by Osteogenesis Imperfecta, even though some variability in the clinical outcome has been reported (52) (Figure 1B).

Clinical case series and a recent analysis of the FREEDOM and FREEDOM Extension Trials about osteoporosis treatment with the anti-RANKL antibody have pointed to an increased risk of multiple vertebral fractures after denosumab discontinuation due to a rebound in bone resorption (53, 54), thus raising a note of caution. In an attempt to identify potential alternative antiresorptive therapies, scientific interest about natural compounds possibly interfering with the RANKL-RANK axis (e.g., flavonoids, alkaloid compounds, triterpenoids, polysaccharides as well as monomeric sugars) has been growing exponentially, as demonstrated by the number of publications evaluating this kind of approach (55–58).

In parallel, recent papers pointed to an unexpected osteogenic function of RANKL through (at least) two different, not mutually exclusive mechanisms: an autocrine-paracrine loop activated by RANKL binding to its receptor(s) on MSCs (27); and a reverse signaling elicited by osteoclast-derived RANK-expressing extracellular vesicles, which might induce membrane-RANKL clustering on osteoblasts (59, 60). This might represent an additional means for osteoblast-osteoclast crosstalk. As a perspective, it might be exploited by means of a new drug with two simultaneous activities: dampening of bone resorption by preventing RANKL binding to RANK receptors on the osteoclasts, and stimulating osteogenesis by triggering RANKL signaling in the osteoblasts (Figure 1A).

Actually, the biological relevance of these new findings in the framework of the overall bone homeostasis has to be clearly defined; for the sake of completeness, opposite results have been reported by others (28). Nevertheless, the possibility of an osteogenic function of RANKL is worth further investigations since it could pave the way to the development of new therapeutic strategies, thus fulfilling a medical need.

## RANKL-RANK Axis in the Thymus

The thymus is a primary lymphoid organ responsible for the development of T lymphocytes expressing a T cell repertoire capable of responding to a diverse array of foreign antigens but tolerant to self-antigens (61, 62). Migrant lymphoid progenitors, arising in the liver during embryonic life and in the bone marrow in postnatal life, enter the thymus where they undergo different phases of differentiation throughout a complex journey from the cortical region to the medullary compartment (63). The early phases of thymocyte differentiation strictly depend on stromal derived signals mediated by the interaction of CD4^+^CD8^+^ double positive (DP) T cell precursors with cortical thymic epithelial cells (cTECs) and indirectly by the production of soluble factors (Figure 1C). cTECs foster lineage commitment during the early stages of T cell differentiation (double negative, DN, stage) through the expression of Notch ligand Delta-like 4 (64, 65) and mediate positive selection of DP T cells by presenting a broad array of self-peptides via major histocompatibility complex (MHC) class I and II molecules. This process results in the survival of thymocytes, which migrate into the thymic medulla where T cells are negatively selected to single positive (SP) CD4^+^CD8^−^ and CD8^+^CD4^−^ T cells (66). Mature medullary thymic epithelial cells (mTECs) mediate central tolerance process by expressing the transcriptional coactivator AutoImmune Regulator (AIRE), which drives the expression of self-antigens, including tissue restricted antigens (TRAs) leading to the clonal deletion of autoreactive T cells, while inducing the generation of regulatory T cells (67, 68), and the intra-thymic positioning of X-C Motif Chemokine Ligand 1 (XCL1)^+^ dendritic cells (69).

Various factors modulate the development and maturation of the thymic epithelial compartment, including several signal transducers regulating NF-κB pathway and the NF-κB family member RelB (70–76). Signaling mediated by four receptors of the tumor necrosis factor family [RANK, OPG, CD40, and lymphotoxin (LT) β receptor] acts as important modulator of thymic microenvironment along with the cross talk between thymocytes and TECs (77–79). In addition, the Ets transcription factor family member Spi-B, which was found to be associated with autoimmune phenomena (80), mediates OPG expression via a negative feedback regulatory loop thus limiting the development of mature TECs (81). RANKL is mainly produced by CD4^+^ cells, a small subset of CD8^+^ cells, invariant (Natural Killer T) NKT cells and CD4^+^CD3^−^ lymphoid tissue inducer (LTi) cells (82, 83). Of note, during embryonic life at the initial stages of thymus development, invariant Vγ5^+^ dendritic epidermal T cells (DETCs) and Vγ5^+^ γδ T cells T cells contribute to central tolerance establishment by promoting CD80^−^Aire^−^ mTECs to become CD80^+^Aire^+^ mTECs (84–86) thus supporting a critical role for RANK signaling in the interaction between fetal γδ T cell progenitors and mTECs (87, 88). Of note, these immune cell subsets provide different physiological levels of RANKL and CD40 Ligand (CD40L) during ontogeny. During fetal life, mTEC development is controlled by the expression of RANKL by LTi and invariant Vγ5^+^ DETC progenitors, while after birth is controlled by RANKL and CD40L produced by αβ T Cell Receptor (TCR)^high^ CD4^+^ thymocytes (89).

Transgenic mice expressing Venus, a fluorescent protein to track RANK expression, showed that this receptor is mainly expressed by mTECs at different stages of differentiation (90). Moreover, activated T cells recirculating to the thymus further contribute to the production of RANKL (91). Thus, it is tempting to speculate that the increased production of RANKL may support the skewing toward mTEC lineage, with consequent maturation of T cells leading to the exhaustion of the progenitor pool. These observations might explain the age-related changes observed in thymic epithelium during aging or thymic dysmorphology found in some pathological conditions (92, 93).

Extensive *in vitro* and *in vivo* studies have further confirmed the relevant role of the RANKL-RANK axis in the establishment and maintenance of the central tolerance process. *In vitro* stimulation of fetal thymic organ culture (FTOC) with recombinant RANKL or agonistic anti-RANK antibody results in the upregulation of CD80 and Aire expression by mTECs (87, 94). In parallel, mice deficient in TCRα or murine models with a reduced number of CD4^+^ T cells for instance lacking molecules of the MHC II complex have a dramatic reduction in Aire^+^ cells and decreased mTEC compartment (95, 96). Other molecular players contribute to TEC differentiation and among them a peculiar role is played by the interferon regulatory factor 7/interferon β/ interferon-α/β receptor/signal transducer and activator of transcription 1 (IRF7/IFNβ/IFNAR/STAT1) pathway (97). During embryonic life, the absence of RANK or RANKL severely affects mTEC maturation resulting in the complete loss of Aire^+^ mTECs (87, 94, 98). However, after birth other factors compensate the absence of RANK signaling allowing the maturation of few Aire^+^ mTECs (94). Furthermore, OPG is expressed by mTECs and genetically deletion in mice causes enlargement of the medulla area (82, 90). Overall, these data indicate that the RANKL-RANK axis is essential for the correct differentiation and development of mTECs and for the formation of the thymic medulla and consequent establishment of self-tolerance (Figure 1C). Consistently with the role of RANKL as a potent mTEC inducer and indirectly as a key player in the control of central tolerance, systemic administration of soluble RANKL (sRANKL) can be considered to treat primary or secondary thymic dysfunction (99). Transgenic mice constitutively overexpressing human sRANKL displayed thymic medulla enlargement (100) and increased number of Aire^+^ mTECs (101). Interestingly, during *in vivo* administration of recombinant soluble RANKL (sRANKL) to cure the bone defect in *Rankl*^−/−^ mice, we observed a dramatic effect of the cytokine on thymic architecture (102) further confirming data reported in literature. Pharmacological sRANKL treatment induced expansion of the medulla in *Rankl*^−/−^ mice and increase of Aire^+^ mTECs. Improvement of thymic epithelium resulted in higher frequency of CD4^+^ and CD8^+^ SP and reduction of double positive thymocytes (102). These data suggest that the exogenous administration of RANKL may be a new therapeutic strategy to boost thymic regeneration. In line with this, compelling evidence indicate that upon body irradiation CD4^+^ cells and LTi cells up-regulate RANKL in the early phase of thymic regeneration. Upon tissue damage, RANKL mediates the increased expression of LTα by LTi cells and reduces the expression of pro-apoptotic genes while increases the expression of the B-cell lymphoma-extra large (Bcl-xl) anti-apoptotic gene (103). The administration of RANKL to wild-type animals confirmed its crucial role in thymic recovery by enhancing TECs, thymocyte numbers, and in parallel increasing vasculature. Improved T cell reconstitution is also mediated by the increased expression of adhesion molecules and chemokines, which foster thymus homing of lymphoid progenitors. Remarkably, since RANKL is the master gene of osteoclastogenesis, it is tempting to speculate that the increased osteoclast activity may also boost hematopoiesis and consequent migration of thymic progenitors. Overall, these *in vivo* findings confirm the therapeutic effect of RANKL suggesting its putative use to boost immune reconstitution in transplanted elderly patients or in patients affected by primary thymic epithelial defects (104–106) (Figure 1D). Conversely, transient inhibition of RANKL in murine models indicate its effect on thymic negative selection of self-reactive T cells specific for tumor antigens, and resulting in an improvement of antitumor immune response (107, 108). However, *in vivo* inhibition of RANKL during prenatal life in rats and mice or long-life inhibition after birth did not show gross effects on innate or humoral immune response (109), thus supporting a possible repurposing of denosumab as anti-tumoral agent in combinatorial treatments and extending its use in the clinical arena.

## T Cells and RANKL-RANK Signaling in Bone Pathology

The overall picture described highlighted the importance of the RANKL-RANK axis in the bone and thymus compartments: in the former, RANKL-RANK signaling influences the bone remodeling process regulating bone cells activities; in the latter, it is pivotal in thymic cell development and T cell maturation and functioning.

After maturation, T cells exert their function centrally and in all the other peripheral organs, going back also to the bone. Although T cell levels represent about 3–8% of total nucleated bone marrow cells in homeostatic conditions (110), in pathological settings T cell recruitment from the periphery may occur and induce molecular and metabolic changes in bone cells, contributing to the bone loss phenotype associated with various conditions such as post-menopausal osteoporosis and Rheumatoid Arthritis (RA) (Figure 1B).

In post-menopausal osteoporotic patients an increase in RANKL production by activated T cells (and B cells, too), alone or in combination with TNFα, has been reported (111, 112). A similar finding has been shown in surgically ovariectomized (OVX) pre-menopausal women (113), further confirming the causative link between estrogen deprivation, T cell activation and RANKL-mediated bone loss previously observed in the murine model (114). Accordingly, 17β-estradiol inhibits thymic expansion after OVX in mice and T cell development, and protects against bone loss, while selective estrogen receptor modulators exhibit agonistic activity on bone but do not affect T lymphopoiesis (115). Of note, a study in thymectomized pre-menopausal women showed a drop in T cell counts after surgery, as expected, with enhanced activation and production of osteoclastogenic factors by the remaining T cells (116). On the other hand, the authors of the study hypothesized that the establishment of not clarified compensatory mechanisms could be responsible for maintaining bone density at levels similar to euthymic age-matched controls.

Another example of bone-thymus interplay is RA, a chronic inflammatory autoimmune disease characterized by joint inflammation, involving mainly synovial membranes, and bone and cartilage destruction (117, 118). In this condition, the synovium and articular tissues are highly enriched in inflammatory leukocytes, likely due to cell recruitment in the inflamed tissue (119), sustained by resident stromal cells of mesenchymal origin (120). The inflammatory process in the joints is suggested to enhance bone loss in patients with RA, in particular when Anti-Citrullinated Protein Antibodies (ACPA), Rheumatoid Factor (RF) and anti-Carbamylated Protein Antibodies (anti-CarP) are present (121, 122). Most of the T cells recruited from the circulation are T helper 1 (Th1), Th17, and Treg cells (123), which express C-X-C Motif Chemokine Receptor 3 (CXCR3), CXCR4, C-C chemokine receptor type 5 (CCR5), and CCR6 (mainly on Th17 cells) receptors that permit their entry into the inflammatory site upon attraction by the high levels of chemokines (e.g., CCL20) found in arthritic joints (124–126). The relevant presence of these cells exacerbates bone erosion by osteoclasts located at the interface between the synovial membrane and bone (48). The pathological bone loss is not compensated by osteoblast-repairing activity since this process is inhibited by synovial inflammation (127). Pro-inflammatory cytokines, such as IL-1, IL-6, and more importantly TNFα and IL-17 are produced in the inflamed synovium and strongly induce RANKL production through the activation of NF-κB pathway in synovial cells and T cells, which in turn massively activate osteoclasts (22, 48, 128). In patients with early RA, RANKL plasma levels have been associated with bone destruction and with radiological progression of the disease after 24 months of follow-up (129). Moreover, the combined presence of increased RANKL levels and the positivity for anti-Cyclic Citrullinated Peptide 2 (anti-CCP2) antibodies correlated with a more destructive process. These data were confirmed in a case-control study conducted in RA pre-symptomatic patients, where RANKL plasma levels were higher in pre-symptomatic individuals as compared to control subjects, increased over time until the onset of RA symptoms and were associated with levels of inflammatory cytokines. However, the positivity for ACPA/RF/anti-CarP preceded the rise of RANKL plasma levels (129).

Based on this, preventing bone erosion by targeting RANKL-RANK axis could be an effective strategy for intervention (130). In fact, taking into account that RANKL-RANK axis is a pivotal immune modulator in DC development and function, in memory B cells, Th17, and Treg cells (131), RANKL blockade might modulate the immune response thus contributing to limit pathological bone erosion and joint damage occurring in RA.

In a phase II trial (Denosumab in patients with RheumatoId arthritis on methotrexate to Validate inhibitory effect on bone Erosion -DRIVE- study) on Japanese RA patients treated with methotrexate, denosumab significantly inhibited the progression of bone erosion at 12 months, and preserved the bone mineral density (132). In addition, in a retrospective cohort trial, the decrease of bone erosion in patients treated with denosumab in combination with biological disease-modifying anti-rheumatic drugs (bDMARDs), at 12 months was significantly higher as compared to denosumab alone, with no adverse effects. Therefore, blocking RANKL-RANK signaling in RA patients by the addition of denosumab to conventional treatment agents may represent a potential new therapeutic option for patients to limits RA pathological outcome (Figure 1B).

Importantly, RA is primarily an autoimmune disease, in which defects in central and peripheral T cell tolerance are involved. Altered intra-thymic selection for the removal of autoreactive T cells may have a great impact on the onset of T cell mediated autoimmune disease (133). In the SKG strain murine model of autoimmune arthritis, bearing a spontaneous point mutation in Zeta Chain of T Cell Receptor Associated Protein Kinase 70 (ZAP-70), alterations in αβ TCR signaling in the thymus have been linked to the escape of autoreactive T cells from negative selection, playing an essential role in immune response in the periphery (134). In turn, the onset of RA may be due to impaired peripheral tolerance mechanisms, mainly elicited by Treg cells, in controlling autoreactive T cells (135, 136). In addition, recirculation of peripheral T cells back to the thymus has been described, and the re-entering cells (mainly Treg cells) might alter central tolerance and induce the deletion of thymic antigen presenting cell populations. This could be considered a mechanism for silencing autoreactive T cells in an RA setting where impaired thymic functions are present (133). Whether alteration of this process may be linked to T cell mediated autoimmunity is still not clear and how T cell production in the thymus and their effector functions in the periphery regulate tolerance maintenance needs further investigation from a therapeutic point of view.

Overall, although targeting RANKL-RANK axis in RA with a RANKL antagonist can improve bone and joints pathological features, it remains to be defined whether an effect on central tolerance and autoimmune reactions is achieved too, because of RANKL requirement for the correct thymic development and production of functional T cells.

Finally, interest has recently grown in another field, i.e., regarding the possibility to exploit immune-related mechanisms based on RANKL-RANK signaling in cancer settings for therapeutic purposes (11). In malignancies with enhanced RANKL expression, such as Multiple Myeloma, denosumab alone is well-known to be effective in terms of overall survival and skeletal-related events (137). In different tumor types that usually have low expression of RANKL, denosumab treatment combined with immune check-point inhibitors might lead to a cross-modulation of antitumor immunity (138, 139). The mechanisms proposed are various: denosumab might act on RANKL-expressing tumor infiltrating lymphocytes and relieve their anticancer activity that is otherwise blocked by engagement of the ligand with RANK receptor on cells of the tumor microenvironment (138, 139). Moreover, RANKL antagonists might put a break on central tolerance by transiently inhibiting negative selection in the thymus, resulting in the release of self-specific T cells in the periphery (108). Finally, the activation of reverse-signaling pathways might be proposed (140, 141), in line with mechanisms described in bone (142). At present, all these possibilities require further investigations; their elucidation might shed light on novel therapeutic perspectives.

## Conclusions

The RANKL-RANK axis exerts pleiotropic effects and consistently involves an ever-increasing number of molecular and cellular players. In the bone and thymus compartments, where the crucial role of RANKL signaling was recognized first, novel functions have recently been discovered. This extends our understanding of the basic biology of these tissues and has translational implications in terms of current therapies monitoring. In particular, opposite effects are expected in the case of blocking or activating the RANKL-RANK pathway on bone and immune tolerance: while used as an antiresorptive drug, the anti-RANKL antibody denosumab might have adverse effects on the establishment of central tolerance, which would deserve attention. On the other hand, recent advances might support efforts toward drug repurposing strategies and development of new medicines, based on limitations of those currently available.

## Author Contributions

All authors listed have made a substantial, direct and intellectual contribution to the work, and approved it for publication.

### Conflict of Interest Statement

The authors declare that the research was conducted in the absence of any commercial or financial relationships that could be construed as a potential conflict of interest.

## Abbreviations

ACPA: Anti-Citrullinated Protein Antibodies
AIRE: Auto Immune Regulator
anti-CarP: anti-Carbamylated Protein
anti-CCP2: anti-Cyclic Citrullinated Peptide 2
Bcl-xl: B-cell lymphoma-extra-large
CCL20: C-C Motif Chemokine Ligand 20
CCR5: C-C chemokine receptor type 5
CCR6: C-C chemokine receptor type 6
cTEC: cortical Thymic Epithelial Cell
CXCR3: C-X-C Motif Chemokine Receptor 3
CXCR4: C-X-C Motif Chemokine Receptor 4
bDMARDs: biological Disease-Modifying Anti-Rheumatic Drugs
DETC: dendritic epidermal T cell
DP: Double Positive
FTOC: fetal thymic organ culture
GAGs: glycosaminoglycans
IL-1: Interleukin 1
IL-6: Interleukin 6
IL-17: Interleukin 17
IFNβ: Interferon beta
IFNAR: Interferon-α/β receptor
IRF7: Interferon Regulatory Factor 7
ITAM: Immunoreceptor Tyrosine-based Activation Motif
LGR4: Leucine Rich Repeat Containing G Protein-Coupled Receptor 4
LTα: Lymphotoxin α
LTi: Lymphoid Tissue inducer cells
MAP kinase: Mitogen Activated Protein kinase
MHC: Major Histocompatibility Complex
MSCs: Mesenchymal Stem Cells
mTEC: medullary Thymic Epithelial Cell
NFATc1: Nuclear Factor Of Activated T Cells 1
NF-κB: Nuclear Factor kappa B
OPG: Osteoprotegerin
OVX: ovariectomized
PI3K: Phosphoinositide 3-kinase
RA: Rheumatoid Arthritis
RANK: Receptor Activator of Nuclear Factor kappa B
RANKL: Receptor Activator of Nuclear Factor kappa B Ligand
RF: Rheumatoid Factor
ROS: Reactive Oxygen Species
SP: Single Positive
STAT1: Signal Transducer and Activator of Transcription 1
TCR: T Cell Receptor
Th1: T helper 1 cells
Th17: T helper 17 cells
TNFα: Tumor Necrosis Factor α
TNFR: Tumor Necrosis Factor Receptor
TNFRSF11A: Tumor Necrosis Factor Receptor Superfamily Member 11A
TNFSF11: Tumor Necrosis Factor Ligand Superfamily Member 11
TRAF6: TNF Receptor-Associated Factor 6
TRAIL: TNF-Related Apoptosis Inducing Ligand
TRAs: Tissue Restricted Antigens
Treg: T regulatory cells
vWF: von Willebrand factor
XCL-1: X-C Motif Chemokine Ligand 1
ZAP-70: Zeta Chain Of T Cell Receptor Associated Protein Kinase 70.
